# A Paradigm in Immunochemistry, Revealed by Monoclonal Antibodies to Spatially Distinct Epitopes on Syntenin-1

**DOI:** 10.3390/ijms20236035

**Published:** 2019-11-29

**Authors:** Ian R. D. Johnson, Alexandra Sorvina, Jessica M. Logan, Courtney R. Moore, Jessica K. Heatlie, Emma J. Parkinson-Lawrence, Stavros Selemidis, John J. O’Leary, Lisa M. Butler, Douglas A. Brooks

**Affiliations:** 1 Mechanisms in Cell Biology and Disease Research Group, School of Pharmacy and Medical Sciences, University of South Australia Cancer Research Institute, University of South Australia, Adelaide, SA 5000, Australia; 2 Adelaide Medical School and Freemasons Foundation Centre for Men’s Health, University of Adelaide, Adelaide, SA 5000, Australia; 3 South Australian Health and Medical Research Institute, Adelaide, SA 5000, Australia; 4 Oxidant and Inflammation Biology Group, Chronic Infectious and Inflammatory Diseases Program, School of Health and Biomedical Sciences, RMIT University, Bundoora, VIC 3803, Australia; 5 Department of Pharmacology, Infection and Immunity Program, Biomedicine Discovery Institute, Monash University, Clayton, VIC 3800, Australia; 6 Department of Histopathology, School of Medicine Trinity College Dublin, Dublin 8, Ireland; 7 Molecular Pathology Laboratory, The Coombe Women and Infants’ University Hospital, Dublin 8, Ireland

**Keywords:** syntenin-1, prostate cancer, microtubules, endosomes

## Abstract

Syntenin-1 is an essential multi-functional adaptor protein, which has multiple roles in membrane trafficking and exosome biogenesis, as well as scaffolding interactions with either the actin cytoskeleton or focal adhesions. However, how this functional multiplicity relates to syntenin-1 distribution in different endosome compartments or other intracellular locations and its underlying involvement in cancer pathogenesis have yet to be fully defined. To help facilitate the investigation of syntenin-1 biology, we developed two specific monoclonal antibodies (Synt-2C6 and Synt-3A11) to spatially distinct linear sequence epitopes on syntenin-1, which were each designed to be unique at the six-amino acid level. These antibodies produced very different intracellular staining patterns, with Synt-2C6 detecting endosomes and Synt-3A11 producing a fibrillar staining pattern suggesting a cytoskeletal localisation. Treatment of cells with Nocodazole altered the intracellular localisation of Synt-3A11, which was consistent with the syntenin-1 protein interacting with microtubules. In prostate tissue biopsies, Synt-3A11 defined atrophy and early-stage prostate cancer, whereas Synt-2C6 only showed minimal interaction with atrophic tissue. This highlights a critical need for site-specific antibodies and a knowledge of their reactivity to define differential protein distributions, interactions and functions, which may differ between normal and malignant cells.

## 1. Introduction

Syntenin-1 (also known as syndecan-binding protein 1, Scaffold protein Pbp1, Pro-TGF alpha cytoplasmic domain interacting protein 18 (TACIP18) and Melanoma differentiation–associated protein (MDA-9), was initially cloned by Lin et al. [1]. It plays a critical role in multiple cellular functions, including the regulation of membrane trafficking, cell adhesion and exosome biogenesis [2]. Syntenin-1 can associate with endosome compartments, including Rab5 early endosomes, Rab7 late endosomes, and Rab11 recycling endosomes [3], and these compartments are altered in prostate cancer though changes in endosome gene expression and endosome distribution [4,5,6]. Understanding the roles that syntenin-1 plays in endosome biology is important to determine how this protein supports cancer cell growth and migration [7] and its role in promoting invasion and metastasis in prostate and other cancers [8,9,10]. Syntenin-1 is a specific component of the exosome-biogenesis machinery, which associates with ALIX and syndecan [11], involving interactions on late endosomes. Syntenin-1 also locates with adherens junctions and focal adhesions and is detected in the endoplasmic reticulum and nucleus [12], the latter of which is consistent with its reported roles in regulating transcription [13]. This critical membrane-associated adaptor protein, therefore, serves multiple roles in cell biology and consequently will have different distributions based on its reported functional interactions within different subcellular regions or with specific structures/compartments.

Syntenin-1 has two closely linked PDZ domains, which can interact with receptor proteins and phosphoinositide lipids/cholesterol to control membrane trafficking [14]. While this dual PDZ domain was originally identified as binding to the proteoglycan syndecan, it is now recognised as a promiscuous domain that interacts with many other PDZ proteins, such as the frizzled and vascular endothelial growth factor receptors [14,15]. Syntenin-1 is involved in controlling the recycling and degradation of syndecans and other cargo, such as growth factors and adhesion molecules in an ARF6-dependent manner [16,17]. This capacity to organise receptors and its ability to target selected proteins into multi-protein complexes is important as it is linked to the aberrant signalling that is involved in cell migration, invasion and metastasis in cancer [18,19]. Syntenin-1 is also involved in linking adhesion molecules to the actin cytoskeleton in an Ezrin-dependent manner, which may underpin its important role in cell adhesion, migration and intracellular organisation [20]. Consequently, syntenin-1 is likely to have distinct spatiotemporal distributions based on these different functions.

Immunochemistry is a widely-used technique that can be employed to characterise the distribution of a target protein, but unfortunately, commercial antibodies are often fallible and may not necessarily depict the specific characteristics of their intended target proteins [21,22,23]. Accurately defining the immunochemistry of syntenin-1 is central to characterising the multiple potential roles reported for this adaptor protein. Antibodies to syntenin-1 are commercially available and like most other immunochemical reagents, are often utilised without a full appreciation of their potential interactions. As proof of this principle, we epitope-mapped a commercial antibody raised to a partial recombinant protein sequence of syntenin-1, and herein have demonstrated that this involves multiple epitopes. Further analysis revealed that each epitope had, on average, between 4 and 37 potential sequence identities with other proteins at the six-amino acid level. With this high degree of uncertainty for antibody interaction, we sought to develop specific monoclonal antibodies to linear sequence epitopes on syntenin-1, which were unique to this protein and had no other potential cross interactions at the six-amino acid level. We have investigated the specificity and cellular distribution of two of these specific monoclonal antibodies, designated Synt-3A11 and Synt-2C6, which were raised against spatially distinct epitopes on syntenin-1.

## 2. Materials and Methods

### 2.1. Epitope Mapping of Commercial Antibody

Peptides of 15-mer length were synthesised across the syntenin-1 protein, with a sequence overlap of five amino acids per peptide (Mimotopes Pty Ltd., Clayton, VIC, Australia). Peptides were resuspended in 50% (*v*/*v*) acetonitrile/water and 20 μg/mL of peptide diluted into 250 μL 0.1 M NaHCO_3_ for ELISA plate coating. Peptides were incubated overnight at 4 °C and then washed three times with TBS containing 0.1% Tween^®^-20 (TBST). The ELISA plate wells were blocked in 5% BSA TBST for 1 h at room temperature, and 100 μL of 1 μg/mL antibody diluted in 5% BSA TBST was added to each well and incubated overnight at 4 °C. The unbound antibody was aspirated, and the plates washed three times in TBST followed by detection using HRP conjugated second antibody for 1 h at room temperature before three washes in TBST and HRP detection using 100 μL of ABTS substrate for 20 min at RT. Absorbance was read at 405 nm using a Wallac Victor plate reader (PerkinElmer Pty Ltd., Glen Waverley, VIC, Australia).

### 2.2. Antibody Design

**Figure 1:**
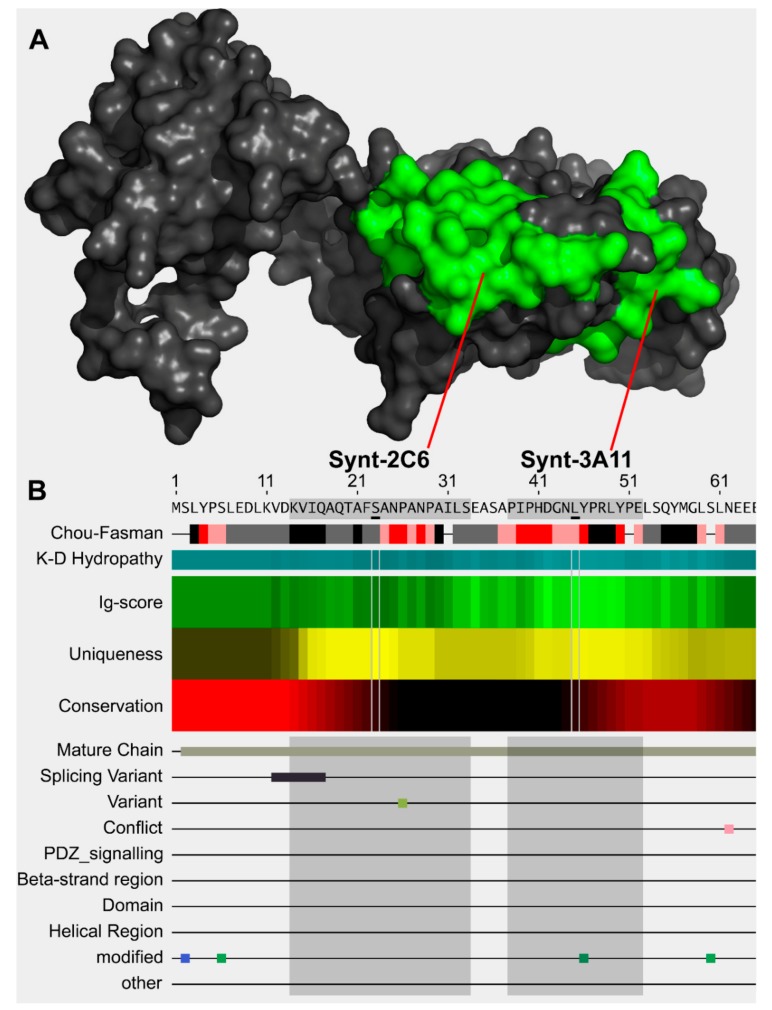
Model of syntenin-1 protein and sequence of syntenin-1 N-terminus epitopes. (**A**) Model of syntenin-1 protein using reference model 1N99 and Phyre^2^, showing spatially distinct unique epitopes of Synt-2C6 and Synt-3A11 (green). (**B**) Selection of unique epitopes using AbDesigner [24] that had no cross-reactivity at the six-amino acid level.

The specific epitopes on syntenin-1 were designed with the aid of AbDesigner [24] and Phyre^2^ [25] to model a complete protein structure and to define spatially separated targets (Figure 1A). To minimise antibody cross-interactivity, we selected unique syntenin-1 epitopes that did not have linear sequence matches with other proteins at the six-amino acid level (Table 1; Figure 1B). Antibody production was outsourced to GenScript for GLP standard production (Piscataway, NJ, USA) and produced in BALB/c mice.

### 2.3. Commercial Antibody Reagents

Primary antibodies used for immunofluorescence included: β-tubulin (1:50; #2146 Cell Signaling Technology, Inc., Danvers, MA, USA), Rab5 (1 μg/mL; Abcam Pty. Ltd., Cambridge, UK, #ab18211), Syndecan-1 (1:200; Santa Cruz Biotechnology Inc., Dallas, TX, USA, #sc5632), Rab27 (1:100; Abcam #ab223044), Rab4 (1:500; Abcam ab109009), Rab11 (1 μg/mL; Abcam ab180778).

The donkey anti-mouse HRP-conjugated secondary antibody (#AP192P, Merck Millipore Pty Ltd., VIC, Australia) was used at 1:10,000 dilution for both Western blots and peptide ELISAs. Immune fluorescence was performed using anti-mouse Alexa Fluor^®^ 488 (1:500; Life Technologies Pty Ltd., Mulgrave, VIC, Australia).

### 2.4. Cell Lines and Culture Conditions

The non-malignant prostate cell lines PNT1a and PNT2 and prostate cancer cell lines 22RV1 and LNCaP (clone FCG) were obtained from the European Collection of Cell Cultures via CellBank Australia (Children’s Medical Research Institute, NSW, Australia). The DU-145 prostate cancer cell line was obtained from the American Type Culture Collection (ATCC; Manassas, VA, USA). Cell lines were cultured in 75 cm^2^ tissue culture flasks and maintained in Roswell Park Memorial Institute (RPMI) 1640 culture medium (Sigma Aldrich Pty Ltd., Castle Hill, NSW, Australia), supplemented with 10% foetal calf serum (Hyclone; In Vitro Technologies Pty Ltd., VIC, Australia) and 2 mM L-glutamine (Sigma Aldrich). Cells were incubated at 37 °C with 5% CO_2_ in a Sanyo MCO-17AI humidified incubator (Sanyo Electric Biomedical Co., Ltd., Osaka, Japan). Cells were cultured to approximately 90% confluence before passage; by washing with sterile PBS (Sigma Aldrich., Castle Hill, NSW, Australia), trypsin treatment (TrypLE™ Express; Thermo Fisher Scientific Australia Pty Ltd., Mulgrave, VIC, Australia) to dissociate the cells from the culture surface, and then resuspension in supplemented culture medium.

### 2.5. Preparation of Cell Extracts and Conditioned Culture Media for Protein Detection

At ~80% confluence, the cell culture media was aspirated, cells washed once with PBS and detached with TrypLE™ Express. TrypLE™ was neutralised with 1% serum and cells pelleted by centrifugation at 200× *g* for 5 min at room temperature. Supernatant was aspirated, and the pellet washed with PBS followed by a further centrifugation step at 200× *g* for 5 min at room temperature. Cell pellets were stored at −80 °C until required. Cell lysate was prepared by resuspending the cell pellet in 800 µL of 20 mM Tris (pH 7.0) containing 500 mM sodium chloride and 2% (*w*/*v*) SDS and protease inhibitors (Sigma Aldrich). The cell lysate was passaged six times through a 26-gauge needle followed by sonication for one minute at a power of 100 watts (SONICA Q-500; Qsonica, LLC., CT, USA). Total protein from the cell extracts was quantified using a bicinchoninic acid assay method, according to the manufacturer’s instructions (Micro BCA kit, Pierce, Rockford, IL, USA). Samples were quantified using a Wallac Victor™ optical plate-reader and Workout software v2.0 (PerkinElmer Glen Waverley, VIC, Australia), using a 5-point parameter standard curve. Cell lysates were stored at −20 °C until required.

### 2.6. Western Blotting

Ten micrograms of total cell protein from whole-cell lysates was heat-denatured (5 min at 95 °C in NuPAGE^®^ LDS Sample Buffer and reducing agent), then electrophoresed at 130 V for 1 h using pre-cast gels in an XCell SureLock Mini-Cell system (Life Technologies). The protein was then transferred to polyvinylidene difluoride membranes (0.2 μm Polyscreen^®^, PerkinElmer). The transfer membranes were blocked for 1 h at RT using a 5% (*w*/*v*) skim milk solution (for clone 3A11) in 0.1% (*v*/*v*) TBS-Tween^®^-20 (blocking solution) or 5% BSA (*w*/*v*; for clone 2C6) and incubated with primary antibody overnight at 4 °C. The membranes were washed in 0.1% (*v*/*v*) TBS-Tween^®^-20 and then incubated with the appropriate HRP-conjugated secondary antibody diluted 1:10,000 in 5% milk block. Membranes were visualised using Novex^®^ ECL chemiluminescent substrate reagent kit (Life Technologies) and ImageQuant™ LAS 4000 imager, software version 1.2.0.101 (GE Healthcare Pty Ltd., NSW, Australia). The intensity of the signal was quantified relative to a reference GAPDH loading control and Amido Black total protein staining (Sigma Aldrich) using AlphaViewSA™ software v3.0 (ProteinSimple Pty Ltd., CA, USA).

### 2.7. siRNA Knock-Down

SMARTpool ON-TARGETplus siRNA was obtained from DharmaCon Inc. (GE Lifesciences, NSW, Australia): SDCBP (6386; DHA-L-008270-00-0005); Non-targeting Pool (DHA-D-001810-10-05); GAPDH Control Pool (DHA-D-001830-10-05). Transfections were performed at 25 nM siRNA concentrations using transfection reagent Lipofectamine^®^ RNAiMax (Life Technologies) for a period of 24, 48 or 72 h in 6-well plates and harvested into RIPA buffer (10 mM Tris; 150 mM NaCl; 1 mM EDTA; 1% Triton X-100). Cells were washed with ice-cold PBS, 200 μL RIPA buffer and inhibitors were added to each well. Cells were scraped and transferred to ice-cold Eppendorf tubes and passed through a 26-gauge needle six times. Cell lysates were centrifuged at 16,000× *g* for 5 min at 4 °C and the supernatant transferred into ice-cold Eppendorf tubes. Cell lysates were stored at −80 °C until required.

### 2.8. ELISA Sandwich Assay

Synt-2C6 capture antibody (5 μg/mL diluted in 0.2 μm filtered 1 × PBS) was used to coat a 96-well Serocluster™ “U” bottom plate (100 μL/well; Costar #2797). The plate was incubated at room temperature for 1 h and subsequently at 4 °C overnight. After incubation, wells were washed in triplicate by adding 180 µL of 1 × TBST (TBS with 0.05% Tween). Wells were then blocked with 250 µL of TBST containing 1% BSA (Sigma Aldrich #A9647) and incubated at room temperature for 1 h. Syntenin-1 purified recombinant protein was serially diluted into a blocking buffer in twofold dilution ratios (1:1024 to 1:262,144). 100 µL of each dilution was added in triplicate. Plates were incubated at room temperature for 1 h. Washing steps were performed, as previously stated. After washing, the Synt-3A11 biotinylated antibody was diluted to 0.125 μg/mL in a blocking buffer, added to each well and incubated at room temperature for 1 h. After washing, 100 µL of streptavidin-HRP (diluted 1:20,000 in blocking buffer) was added before incubation at room temperature for 1 h. Washes were performed six times before adding 100 µL of TMB substrate (Thermo Fisher Scientific #34029) to each well and incubating at RT on a plate shaker at 700 rpm for 20 min. The substrate reaction was stopped by adding 40 µL of 2 M H_2_SO_4_. The optical absorbance of each well was measured using a plate reader (PerkinElmer EnSpire^®^ Multimode Plate Reader #2300-0000). The absorbance values were determined from the background subtracted from the signal at 450 nm.

### 2.9. Immunofluorescence

Cells (~1 × 10^5^ cells/mL) were cultured for 48 h on 13 mm #1.5 glass coverslips (*n* ≥ 3 for each cell line). The culture media was aspirated, and the cells fixed with 4% (*w*/*v*) formaldehyde (Sigma Aldrich) in PBS for 10 min at RT. Cells were incubated with a blocking solution and permeabilised concurrently with 5% (*w*/*v*) bovine serum albumin containing 0.05% Saponin in PBS for 2 h at RT and agitated by rocking slowly. Cells were incubated with the primary antibody, diluted in 5% BSA, overnight at 4 °C. Cells were washed with three five-minute PBS washes and fluorophore-conjugated secondary antibody, diluted in 5% BSA in PBS, added to the cells and incubated for 1 h in the dark, at RT. Unbound antibody was removed by three five-minute PBS washes, coverslips immersed in dH_2_O and mounted with ProLong^®^ Diamond Antifade Reagent containing DAPI nuclear stain (Life Technologies). Confocal microscopy was performed using a Nikon A1+ laser scanning microscope and associated software (NIS-Elements 4.2; Nikon, Japan). Laser lines of 403 nm and 488 nm were utilised for DAPI and Alexa Fluor^®^ 488 fluorescence, respectively. Images were obtained at a resolution of 0.1 μm/px using a 60× objective lens with numerical aperture 1.4 and refractive index 1.515 and scanner zoom 2 and pinhole 1.2 AU; the pixel resolution was 1024^2^. Semi-automated quantification of Synt-2C6 and endosome cofluorescence was performed from z-stacks with a z-depth of 0.175 μm using NIS-Elements 4.2 software. Imaging of β-tubulin (1:50 dilution; #2146 Cell Signaling Technology, Inc., Danvers, MA, USA) and Synt-3A11 (2 μg/mL) was performed using Zeiss LSM880 confocal microscope with Airyscan (Carl Zeiss AG, Germany). Images were exported as greyscale 16-bit TIFF files and representative figures created using Adobe^®^ Photoshop^®^ CC (2016; Adobe Systems Inc., CA, USA).

### 2.10. Nocodazole Treatment

PNT2 cells were cultured as above. At 48 h post-seeding, cells were treated by replacing the culture media with 350 μL RPMI containing 4, 20 or 100 μM Nocodazole prepared in DMSO. Cells were incubated for 30 min at 37 °C before fixing in 4% PFA and processing for immune fluorescence as above.

### 2.11. Immunohistochemistry

Prostatectomy samples (*n* = 4) were acquired from the Peter MacCallum Cancer Centre (Melbourne, Australia). Matched human non-malignant and malignant prostate cancer tissue sections (2 µm) were mounted on Superfrost™ Ultra Plus^®^ slides (Menzel–Gläser GmbH, Braunschweig, Germany) and heated at 60 °C for 1 h before storage at 4 °C. Sections were then dewaxed in xylene, rehydrated in a graded series of ethanol and incubated in 3% H_2_O_2_ in TBS for 5 min at RT. Heat-induced epitope retrieval was carried out using Tris-EDTA Buffer (10 mM Tris Base, 1 mM EDTA Solution, 0.05% Tween^®^-20, pH 9.0) in a Sharp model R-9270 microwave oven heated on high for 4 min and medium-low for a further 15 min. Sections were cooled in Tris-EDTA to RT using a cooled water bath for 30 min. Sections were incubated with the primary antibody in antibody diluent (Synt-3A11 0.13 ng/mL, Synt-2C6 0.26 ng/mL; Dako Australia Pty Ltd., NSW, Australia), for 1 h at room temperature in a humid chamber, followed by incubation with the appropriate DAKO EnVision™ + System (Dako Australia Pty Ltd., NSW, Australia) as per manufacturer’s instructions. The tissue sections were then counterstained with Ehrlich’s haemotoxylin, rinsed in water, dehydrated in ethanol and a coverslip applied over DPX mounting media (Merck Millipore Pty Ltd., VIC, Australia). Images were obtained by scanning the slides using a Zeiss Axio Scan.Z1 in brightfield mode, using 40x objectives (Zeiss, Jena, Germany).

### 2.12. Ethics

Approval for the use of human prostate tissue sections was obtained from the Ethics Committees of the University of South Australia (Application ID 0000036070; approval date: 25 November 2016) and the University of Adelaide. Informed consent was obtained from all subjects. All experiments were performed in accordance with the guidelines of the National Health and Medical Research Council (Australia).

### 2.13. Data Availability

The datasets generated during and/or analysed during the current study are available from the corresponding author on reasonable request.

## 3. Results

### 3.1. Significant Epitope Cross-Reactivity for Commercial Antibodies to Syntenin-1

The commercially available polyclonal antibodies to syntenin-1 displayed multiple epitopes and a high potential for cross-reactivity with other proteins at each antigenic site. For example, the epitopes for the syntenin-1 polyclonal antibodies were blast-searched, and each had significant potential for cross-reactivity, with between 11 to 37 proteins identified with linear sequence identity at the six-amino acid level (Table 2). We also examined the potential cross-reactivity of a commercial monoclonal antibody to syntenin-1 by epitope mapping and protein blast-searching, which indicated potential interactions with four other proteins at the six-amino acid level (Table 3). To demonstrate the potential cross-reactivity of these commercial antibodies, we tested three antibodies by Western blotting and immunofluorescence. Antibody ab154940 produced no signal by Western blotting or immunofluorescence, and an additional vial requested from the company also produced no detectable signal. Western blotting with antibody ab62530 displayed a molecular species at ~26 kDa in all lysates (Figure S1), and there was a strong interaction with a molecular species at ~130 kDa in 22RV1 and LNCaP cell lysates. The antibody ab62530 also detected a ~24 kDa molecular species in DU-145 lysates and a ~37 kDa molecular species in LNCaP lysates. Cross-reactivity was also observed on Western blots probed with ab19903 (Figure S1), with the detection of a 32 kDa molecular species in all lysates, but additional molecular species of ~25, 22, 18 and 12 kDa. This antibody interaction was previously ‘knock-out validated’, but the product specification sheet also highlighted a cross-reacting protein species at greater than 70 kDa.

Testing by immunofluorescence confirmed that there was no detectable reactivity with ab154940. There was diffuse staining of ab19903 in prostate cancer and non-malignant cells (Figure S2) with similar staining intensities in PNT1a, DU-145 and LNCaP cells. PNT2 cells had less fluorescence staining with ab19903, whilst 22RV1′s displayed increased immunofluorescence. By comparison, ab6250 displayed more defined puncta, distributed across the cytoplasm of cells and increased fluorescence in LNCaP cells (Figure S2).

### 3.2. Immune Fluorescence Demonstrated a Different Cellular Distribution for the Two Syntenin-1 Mono-Specific Monoclonal Antibodies in Prostate Cells

**Figure 2:**
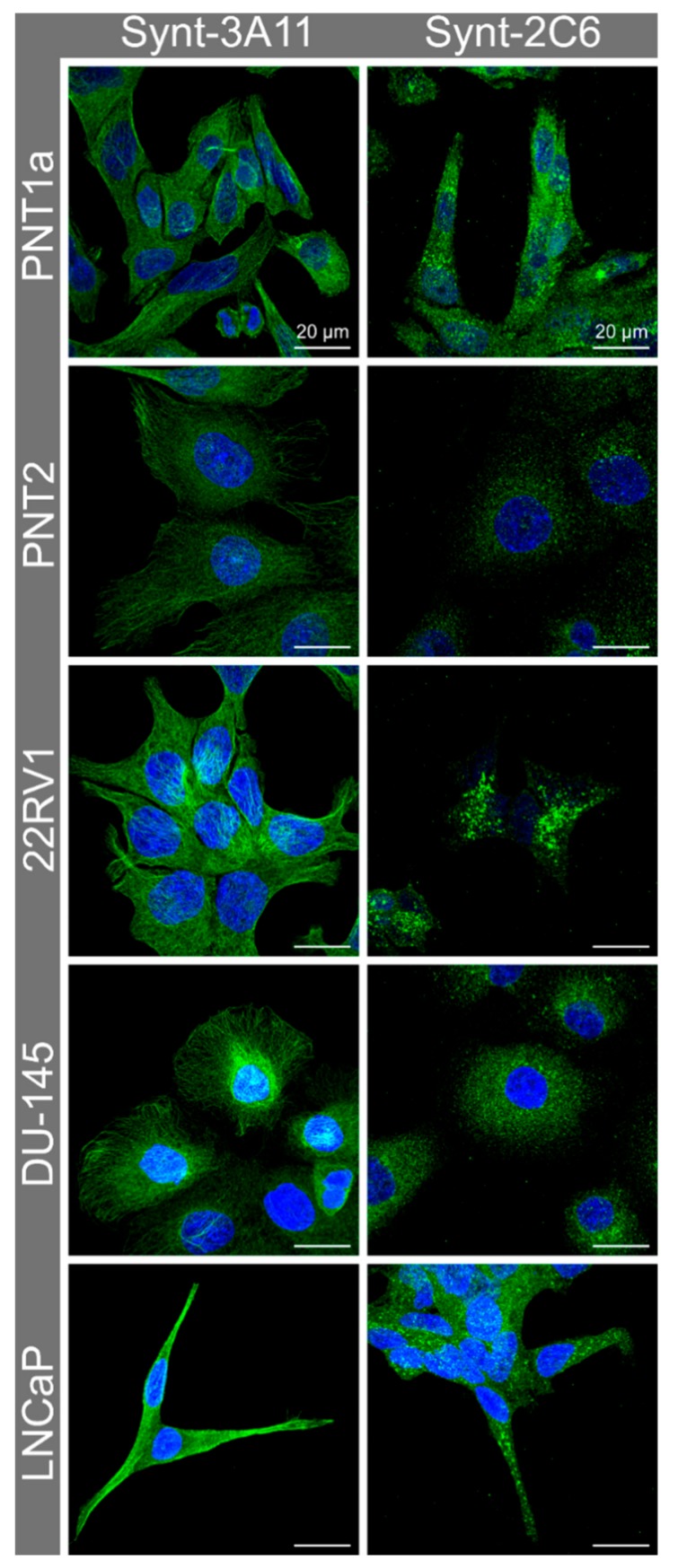
Confocal micrographs of syntenin-1 demonstrate different cellular distribution.

In non-malignant PNT1a and PNT2 cells and malignant 22RV1 and DU-145 cells, the Synt-3A11 monoclonal antibody produced a fibrillar staining pattern, which was consistent with a distribution on either actin filaments or microtubules (Figure 2 and Figure S3). This fibrillar staining pattern was less evident in LNCaP cells, which may relate to the elongated morphology of these cells (Figure 2). In contrast, the Synt-2C6 monoclonal antibody produced a punctate vesicular staining pattern in all the non-malignant and malignant cells tested, which was consistent with an interaction with intracellular compartments (Figure 2).

Syntenin-1 immunofluorescence was performed on non-malignant (PNT1a, PNT2) and prostate cancer cell lines (22RV1, DU-145, LNCaP) using mono-specific monoclonal Synt-3A11 and Synt-2C6 antibodies and showed two spatially distinct epitopes in prostate cell lines. Cells were cultured for 48 h before fixation and permeabilisation, probed with Synt-2C6 or Synt-3A11 primary antibody (1 μg/mL) overnight at 4 °C and detected using 1:1000 anti-mouse Alexa Fluor^®^ 488.

**Figure 3:**
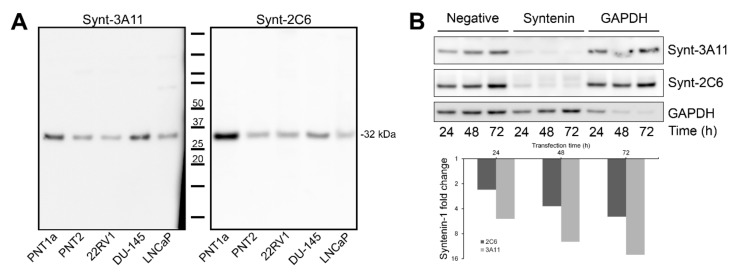
Both the Synt-3A11 and Synt-2C6 monoclonal antibodies interacted specifically with a 32 kDa molecular species of Syntenin-1. (**A**) Ten micrograms of total cell protein from whole-cell lysates of non-malignant PNT1a and PNT2, and 22RV1, DU-145 and LNCaP cancer cell lines were analysed by Western blotting using Synt-3A11 or Synt-2C6 (1 μg/mL) antibodies. Full-length blots are shown. (**B**) siRNA (non-specific control, *SDCBP* or *GAPDH*) was transfected into PNT2 cells for 24, 48 or 72 h and Western blotting performed on 10 μg total protein from whole cell lysate. Detection was performed using 1:10,000 anti-mouse HRP, Novex^®^ ECL chemiluminescent substrate and ImageQuant™ LAS 4000 imager. Detection using Synt-3A11, Synt-2C6 were performed using separate SDS-PAGE gels. Uncropped Western blots are contained within Supplementary Materials.

**Figure 4:**
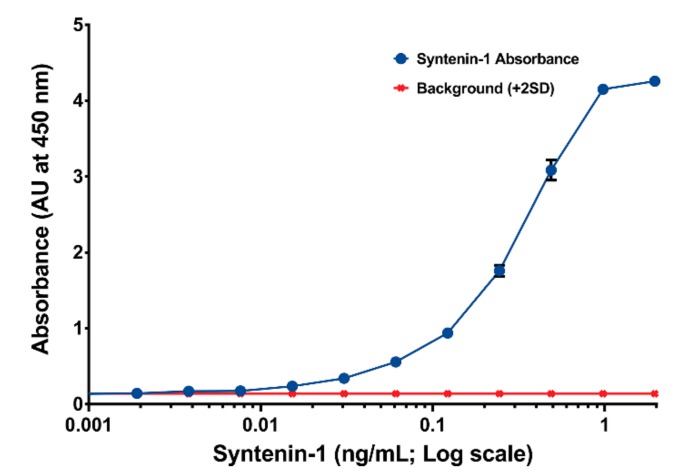
A sandwich ELISA detected purified syntenin-1 protein to ≤4 pg/mL (0.4 pg/well).

Both Synt-3A11 and Synt-2C6 monoclonal antibodies interacted with the same syntenin-1 protein Western blotting with either the Synt-3A11 or the Synt-2C6 monoclonal antibodies showed an interaction with a single distinct 32 kDa molecular species in each of the malignant and non-malignant prostate cell lines tested (Figure 3A), which was the molecular weight expected for syntenin-1 [12]. Using PNT2 cells transfected with *SDCBP* siRNA, we confirmed that both antibodies showed evidence of reduced syntenin-1 protein expression by reduced immune reactivity on Western blots (Figure 3B). Densitometry of the Synt-2C6 signal showed a two-fold reduction within the first 24 h, and a 4-fold reduction after 48 or 72 h (Figure 3B). The signal from Synt-3A11 had a 4-fold reduction at 24 h and 8- and 16-fold reduction at 48 or 72 h (Figure 3B). These knock-downs confirmed that both monoclonal antibodies were detecting the syntenin-1 protein. To further confirm that these two monoclonal antibodies were detecting the same protein, a sandwich ELISA assay was constructed using one of the monoclonal antibodies to capture the target protein and one for detection (Figure 4). This sandwich ELISA with purified syntenin-1 protein had a detection limit of ≤4 pg/mL (0.4 pg/well), using Synt-2C6 as the capture antibody (5 μg/mL), and Synt-3A11 as a biotin-conjugated detection antibody (0.125 μg/mL). This confirmed the specificity of the monoclonal antibodies for syntenin-1, and that the two epitopes were on the same protein. This also confirmed that the epitopes were sufficiently separated to avoid steric hindrance, enabling simultaneous interaction of the antibodies with the same syntenin-1 protein.

A sandwich ELISA was performed using Synt-2C6 as the capture antibody (5 μg/mL), with purified protein captured for 1 h. A biotin-conjugated Synt-3A11 was used as detection antibody (0.125 μg/mL) and streptavidin HRP (1:20,000) used to detect and react UltraTMB^®^ substrate. Absorbance (blue line) was determined at 450 nm from plates read using an Enspire plate reader.

### 3.3. Nocodazole Treatment Confirmed that Syntenin-1 Can Associate with Microtubules

**Figure 5:**
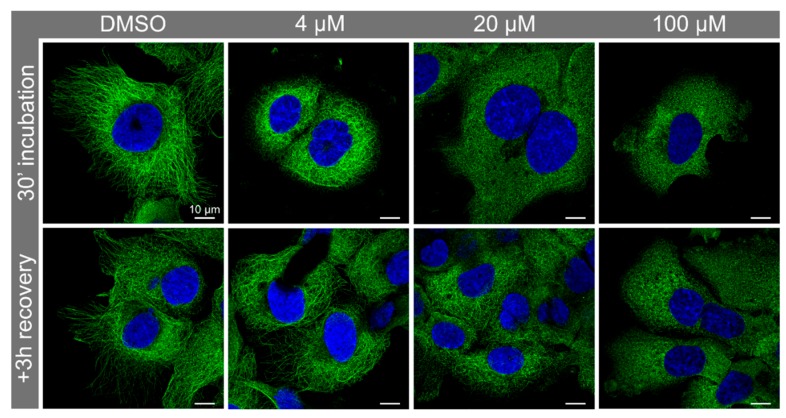
Nocodazole treatment and recovery revealed varying degrees of microtubule disruption visualised by Synt-3A11 immunofluorescence.

**Figure 6:**
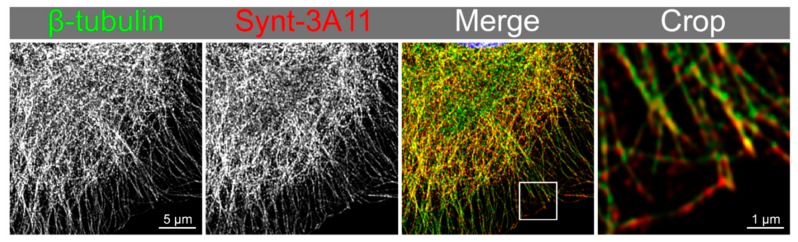
Synt-3A11 colocalises on microtubules depicted with β-tubulin.

**Figure 7:**
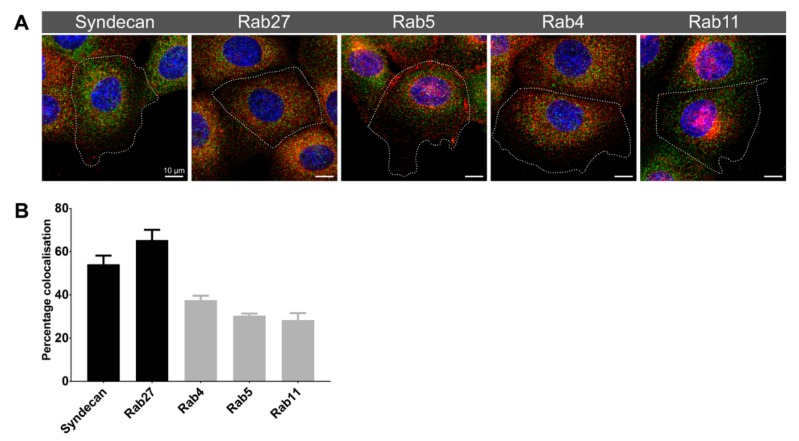
Synt-2C6 localised predominantly with syndecan-1 and Rab27. (**A**) Maximum intensity projections from confocal micrographs of Synt-2C6 (green) and endosome-associated protein (red) immunofluorescence in PNT2 cells. (**B**) Percentage colocalisation of Synt-2C6 with endosome markers in PNT2 cells (*n* = 5) quantified from reconstructed z-stacks.

We used Nocodazole to disrupt microtubules in PNT2 cells to determine if the Synt-3A11 antibody was detecting these cytoskeletal structures. Altering the concentration of Nocodazole revealed variable amounts of microtubule disruption at 30 min (Figure 5), and after a concentration of 20 μM, microtubule re-polymerisation was observed at 3 h after toxin removal (Figure 5). Immunofluorescence analysis of syntenin-1 with Synt-3A11 together with β-tubulin confirmed that there was co-location with microtubules (Figure 6). The amount of Synt-2C6 co-locating with endosome associated proteins was quantified by immunofluorescence (Figure 7A), with 54% of Synt-2C6 colocalising with syndecan; 65% colocalising with Rab27 (Figure 7B). Synt-2C6 collocating with syndecan-1 and Rab27 had a Pearson’s coefficient of approximately 0.6, which was a moderate correlation. There was a lesser amount of Synt-2C6 collocating with Rab5 (30%), Rab4 (38%) and Rab11 (28%; Figure 7B).

PNT2 cells were treated with Nocodazole (4, 20 and 100 μM) for 30 min before recovery using untreated media for 3 h. Microtubule disruption and re-polymerisation were observed by immunofluorescence using Synt-3A11 (1 μg/mL).

Maximum-intensity projection from Airyscan confocal imaging of β-tubulin (green) with syntenin-1 (Synt-3A11 2 μg/mL; red) in PNT2 cells that had been fixed and permeabilised. Colocalisation is depicted in yellow (merge and crop).

### 3.4. Immunohistochemistry with Synt-3A11 and Synt-2C6 Monoclonal Antibodies on Prostate Tissue

**Figure 8:**
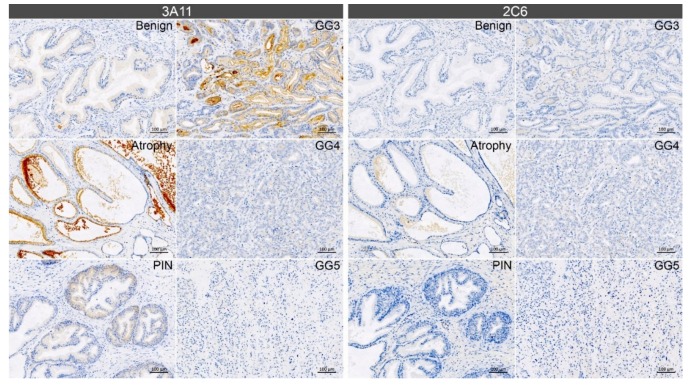
Synt-3A11 defined the luminal membrane of prostate glands. Representative images from immunohistochemistry performed using Synt-3A11 (0.13 ng/mL) and Synt-2C6 (0.26 ng/mL) antibodies on matched human non-malignant and malignant prostate cancer tissue sections. GG—Gleason grade. Scale bars: 100 µm.

The differential distribution patterns for the two monoclonal antibodies Synt-3A11 and Synt-2C6 in cultured cells prompted us to use these antibodies to examine different aspects of syntenin-1 biology in non-malignant and malignant regions of prostate tissue from cancer patients (Figure 8; Figure S4). Interestingly, Synt-3A11 produced stronger staining than Synt-2C6 in benign, atrophic and prostatic intraepithelial neoplasia (PIN) tissue (Figure 8). In the atrophic regions, strong Synt-3A11 staining was evident in luminal epithelial cells and in glandular secretions. In the early stages of prostate cancer (Gleason grade 3), a diffuse staining pattern was observed in the luminal epithelial cells but with strong apical staining. Notably, there was minimal Synt-3A11 staining in more advanced Gleason grade 4 and 5 cancer tissue. This staining indicated an association of Synt-3A11 with the luminal membrane and suggested that microtubule-associated syntenin-1 may be upregulated in early-stage prostate cancer, but downregulated in higher-grade cancer.

## 4. Discussion

Syntenin-1 has multiple interactions, including binding with the cytoplasmic domain of the syndecan family of heparan sulphate proteoglycans [26], plasma membranes, cell-adhesion sites, microtubules and stress microfilaments [12]. It is expected that these associations may mask areas of the syntenin-1 protein structure related to the ligand binding location and steric hindrance. This highlights a potential limitation of visualising intracellular proteins with antibodies that interact at functional sites, for example, using site-specific linear sequence antibody detection reagents. Polyclonal antibodies that are raised against native or long segments of protein tend to have multiple epitope interactions on the target protein, but paradoxically this might not accurately define the target protein due to the potential for cross-reactivity with unrelated proteins; noting that the spatial contact of an antibody can be approximately 15 amino acids [27], but this only requires approximately five or six matching amino acids to have a strong influence on the binding interaction [28]. Consequently, epitope mapping of the natural antigenic sites on a target protein may not necessarily provide ideal sites for antibody production, despite the antibody having a high affinity for specific peptides, as there can be the potential for cross-reactivity due to the relatively high probability of any six amino acid linear sequence occurring throughout the human proteome. In many instances, even a monoclonal antibody to a short linear sequence epitope can also have multiple potential interactions with other proteins due to sequence identity or similarity e.g., [21,22,23], and commercial antibodies may also be a blend of monoclonal antibodies with multiple specificities [29]. Poorly characterised antibodies can waste millions of dollars and years of research due to misinterpretations, as recently found with oestrogen receptor beta, where only one of thirteen anti-ERβ antibodies specifically targeted the receptor [30].

We have shown that with careful design, monoclonal antibodies can be crafted with tools such as AbDesigner [24] to specifically interact with a unique linear epitope at the six-amino acid level; potentially providing optimal antibody specificity. Fundamental cell biology relies heavily on the accurate definition of the spatiotemporal dynamics of target molecules, and this may involve a trade-off between specificity and interactivity for detection reagents. However, the use of multiple site-specific protein detection reagents may still provide reliable reporting on the biology of the target protein. Multiple monoclonal antibodies to differing epitopes can, therefore, potentially delineate biology more specifically than polyclonal antibodies.

Limitations in detection remain where the affinity of antibody interactions is affected, for example, as a result of structurally or chemically modified functional sites, masking of interaction sites or from protein conformational changes. The two monoclonal antibodies, Synt-2C6 and Synt-3A11, highlight the potential for specific antibodies to display differential detection of syntenin-1, based on its functional engagement.

The C-terminal region of syntenin-1 has previously been observed to associate with microfilaments, requiring a short peptide sequence at positions 92–103 [12]. Syntenin-1 is required for the polymerisation of actin [31] and rearrangement of the actin cytoskeleton in extra-embryonic tissues [32]. Syndecan-1 is a potential binding partner for syntenin-1 and is also known to associate with microtubules [33]. Thus, the binding of syntenin-1 to endosomes may alter the protein structure or mask the Synt-3A11 epitope limiting the antibody interaction with microtubules. Conversely, microtubule binding may mask the Synt-2C6 epitope limiting its detection on endosomes while exposing the Synt-3A11 epitope. These specific sites on syntenin-1 may, therefore, provide a functional measure of the dynamic relationship of, for example, syntenin-1 and syndecan-1 binding and functional interactions with microtubules and endosomes.

We have also investigated these functional interactions in the context of disease pathogenesis to visualise functional differences in syntenin-1 in malignant compared to non-malignant tissue. The epitope detected by Synt-3A11 could be used to visualise malignant and non-malignant pathology in prostate cancer tissue, showing greater staining in atrophic tissue and early cancer cells, particularly at luminal borders of prostate cancer tissue. However, the Synt-2C6 epitope showed very limited staining in malignant tissue, which might be consistent with altered conformation or protein association, and increased syntenin-1 microtubule association. These specific epitope detection patterns may, therefore, reflect the differential involvement of syntenin-1 in the cancer process with increased expression in early cancer and or specific microtubule related function. This warrants further exploration of syntenin-1 expression patterns in cancer, and mechanistic studies to define the involvement in cancer establishment and progression. Syntenin-1 over-expression can enhance cancer cell migration via the activation of focal adhesion kinase-JNK or focal adhesion kinase-Akt signalling [9], and in turn, influence the aggressiveness of prostate cancer cells [34] and effectively contribute to cancer progression [35]. Interestingly, syntenin-1 is also required for exosome formation, accounting for its detection in focal adhesions and the recruitment of focal adhesion kinases into exosomes in prostate cancer cells [36]. Synt-3A11 may, therefore, be used to dissect the subcellular pathology at critical sites of cancer pathogenesis.

We have defined two antibodies that recognise different functional sites on the syntenin-1 protein and highlight a need for high-quality reagents and an increased understanding of the biology of the protein/antibody interaction, so that accurate interpretation can be made about specificity, distribution and tissue interaction. This is extremely important for precision immunochemistry and an often-overlooked concept, based on the false assumption that an epitope-specific antibody will always detect the protein target. There are precedents for functionally specific antibodies. For example, in prostate cancer detection, Elecsys^®^ free PSA test (Roche Diagnostics Ltd.) measures free PSA to determine the ratio of free/total PSA to more specifically diagnose prostate cancer. ‘Total’ PSA includes the detection of PSA complexed with α1-antichymotrypsin, which is a dominant form in prostate cancer patients. Free PSA is not bound to α1-antichymotrypsin, and antibodies were developed that target the active site, which is usually involved in the binding to this protein (e.g., [37]). Thus, the development of optimal protein-based diagnostic tools requires knowledge of binding partners and target specific protein functions, rather than just global expression changes.

Through careful design of Synt-2C6 and Synt-3A11, the antibodies recognise unique epitopes that are specific to syntenin-1 and provide a potential method to illuminate novel syntenin-1 biology, such as microtubule binding, and provide a method for the detection of changes in this biology during disease onset and progression. Appropriate antibody design to ensure specific target detection is critical for the important interpretations made by immunochemists, cell biologists and pathologists, and has the potential to generate reagents that provide precise spatial, temporal and functional information on a target protein.

## Figures and Tables

**Table 1:** Epitope selection for syntenin-1 monoclonal antibody production.

Clone	Epitope	aa Range	Cross-Reactivity ≥ 6 aa
Synt-2C6	KVIQAQTAFSANPANPAILS	14–33	0
Synt-3A11	PIPHDGNLYPRLYPE	38–52	0

**Table 2:** Potential epitope reactivity for commercial polyclonal antibodies.

	Sequence Range	Matches ≥ 6 aa	Potential Cross-Reactivity
*ab154940*	1–45	37	ANDR, CE042, CENPM, CF211, CLIP2, CTL1, DESP, EPHX4, FNTB, FOXO4, GLSK, HERC5, HERC6, KAT2A, KC1A, KC1AL, KC1D, KC1E, KC1G1, KC1G2, KC1G3, LUZP1, M3K2, MOSC1, NPHP4, NTAL, PO6F1, Q71TU5, Q8IWC0, Q9BZG5, SDCB2, SLN13, TFDP2, TITIN, TTC28, UBE3C, ZCC18
*ab53552*	6–19	11	CF211, CLIP2, DESP, FNTB, HERC5, HERC6, LUZP1, SDCB2, TFDP2, TITIN, UBE3C
*ab62530*	109–158	29	AKA11, B3A3, CAN7, CG063, CK093, CNTP1, DYH3, DYH7, DYST, EAA3, FAT1, GEMI4, HAUS6, MACF1, MACF4, MTCH1, MYO15, PAPL, PDE12, Q5TDC2, Q5VY60, RGS22, SC16A, SDCB2, SV2A, TBCD, VEGFC, VP13B, WDR35

**Table 3:** Cross-reactivity for commercial syntenin-1 monoclonal antibody.

Epitope Reactivity	Range	Matches ≥6 aa	Protein Cross-Reactivity
*HDGNLYPRLYPELSQYMGLS*	41–60	4	CQ047, DYST, EPG5, SDCB2

## Supplementary Materials

Supplementary materials can be found at https://www.mdpi.com/1422-0067/20/23/6035/s1. Figure S1. Western blotting of ab62530 and ab19903 on whole cell extracts, Figure S2. Immunofluorescence on fixed and permeabilised cells using ab62530, ab19903 and ab154940, Figure S3. Additional immunofluorescence staining of prostate cells using Synt-2C6 and Synt-3A11, Figure S4. Additional immunohistochemistry staining of human prostate tissue sections using Synt-3A11 (0.13 ng/mL) and Synt-2C6 (0.26 ng/mL).
