# Supplementary material for: A Paradigm in Immunochemistry, Revealed by Monoclonal Antibodies to Spatially Distinct Epitopes on Syntenin-1
Source: Int J Mol Sci. 2019 Nov 29;20(23):6035. doi: 10.3390/ijms20236035 (PMC6928784; doi:10.3390/ijms20236035)
Supplement: Supplementary file 1 [file ijms-20-06035-s001.pdf]

SUPPLEMENTARY DATA

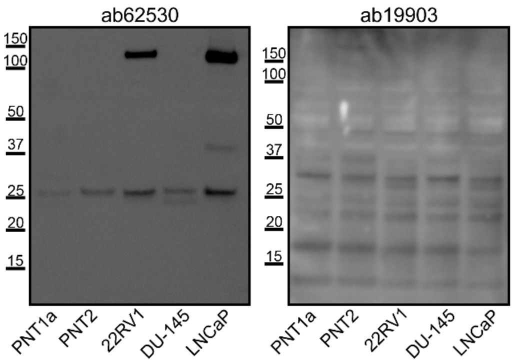

Figure S1. Western blotting of ab62530 and ab19903 on whole cell extracts.

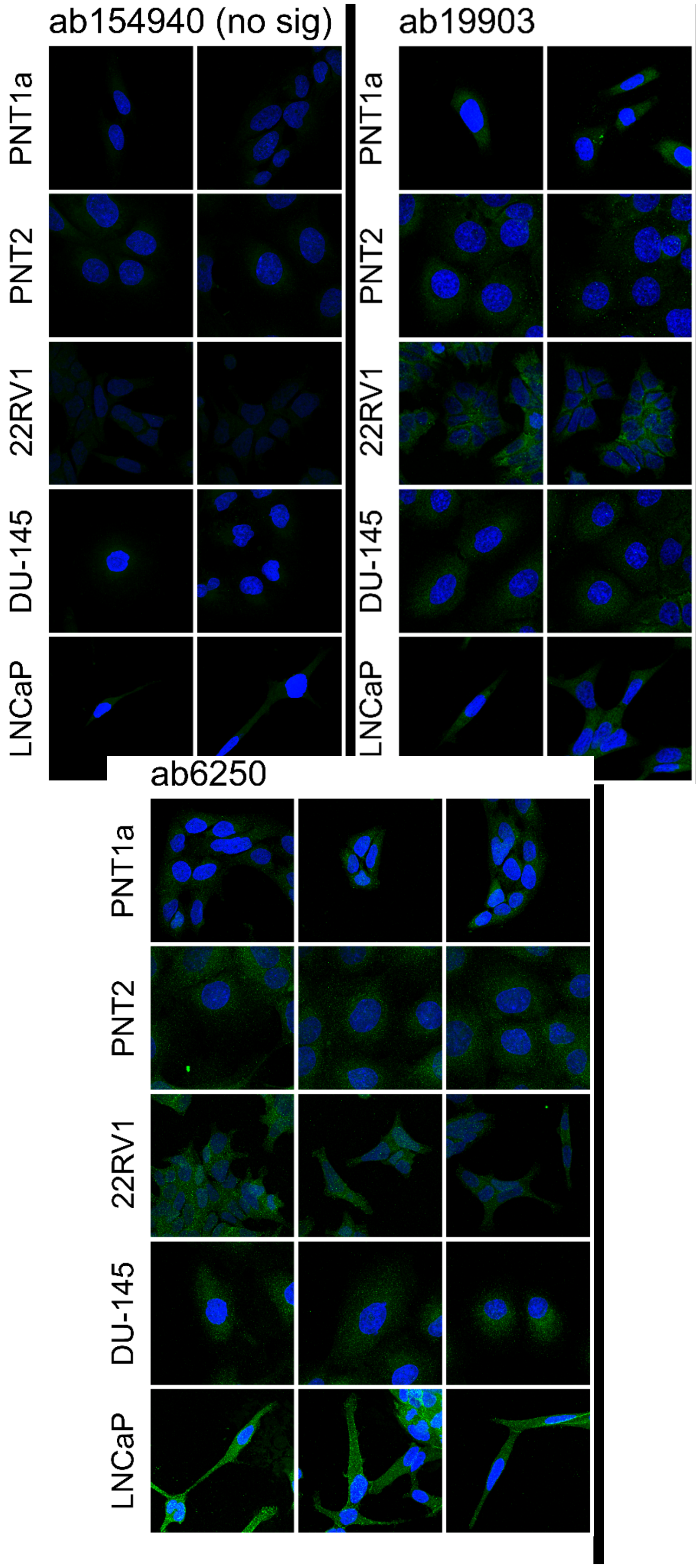

Figure S2. Immunofluorescence on fixed and permeabilised cells using ab62530, ab19903 and ab154940.

2C6

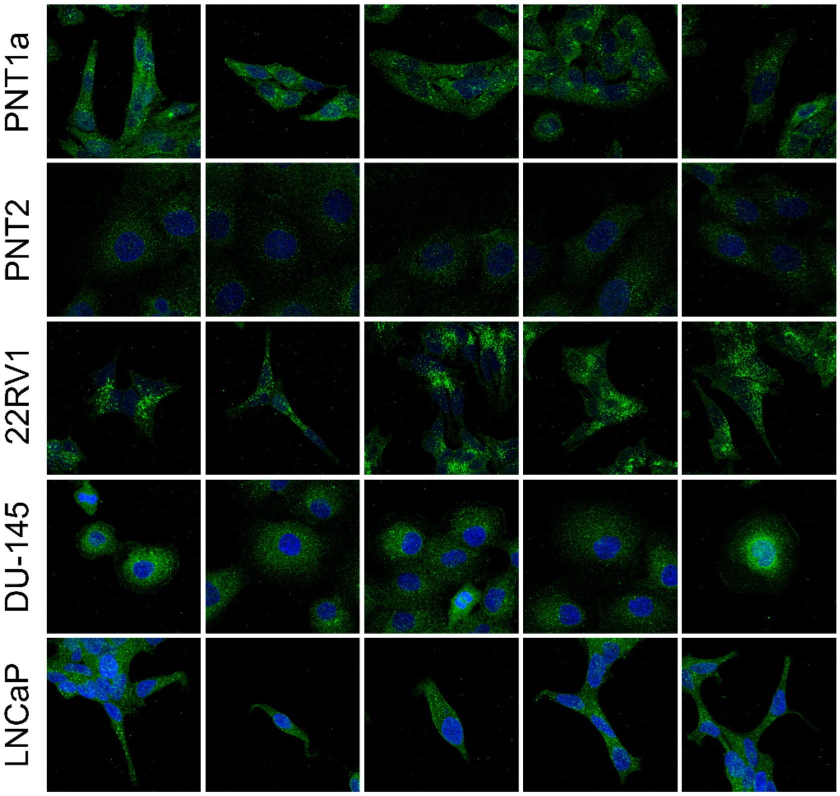

3A11

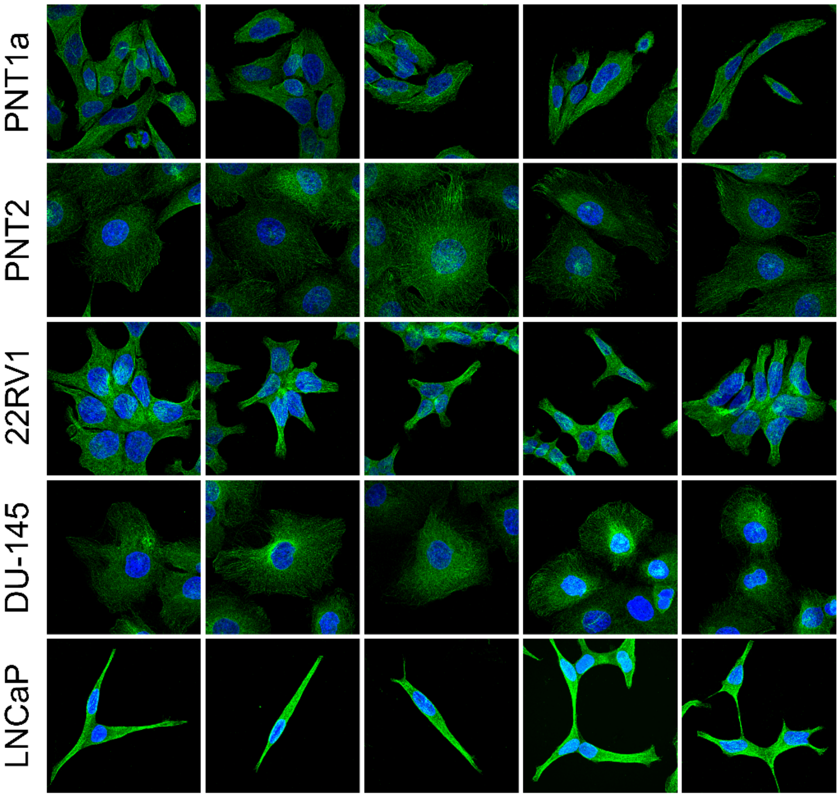

Figure S3. Additional immunofluorescence staining of prostate cells using Synt-2C6 and Synt-3A11.

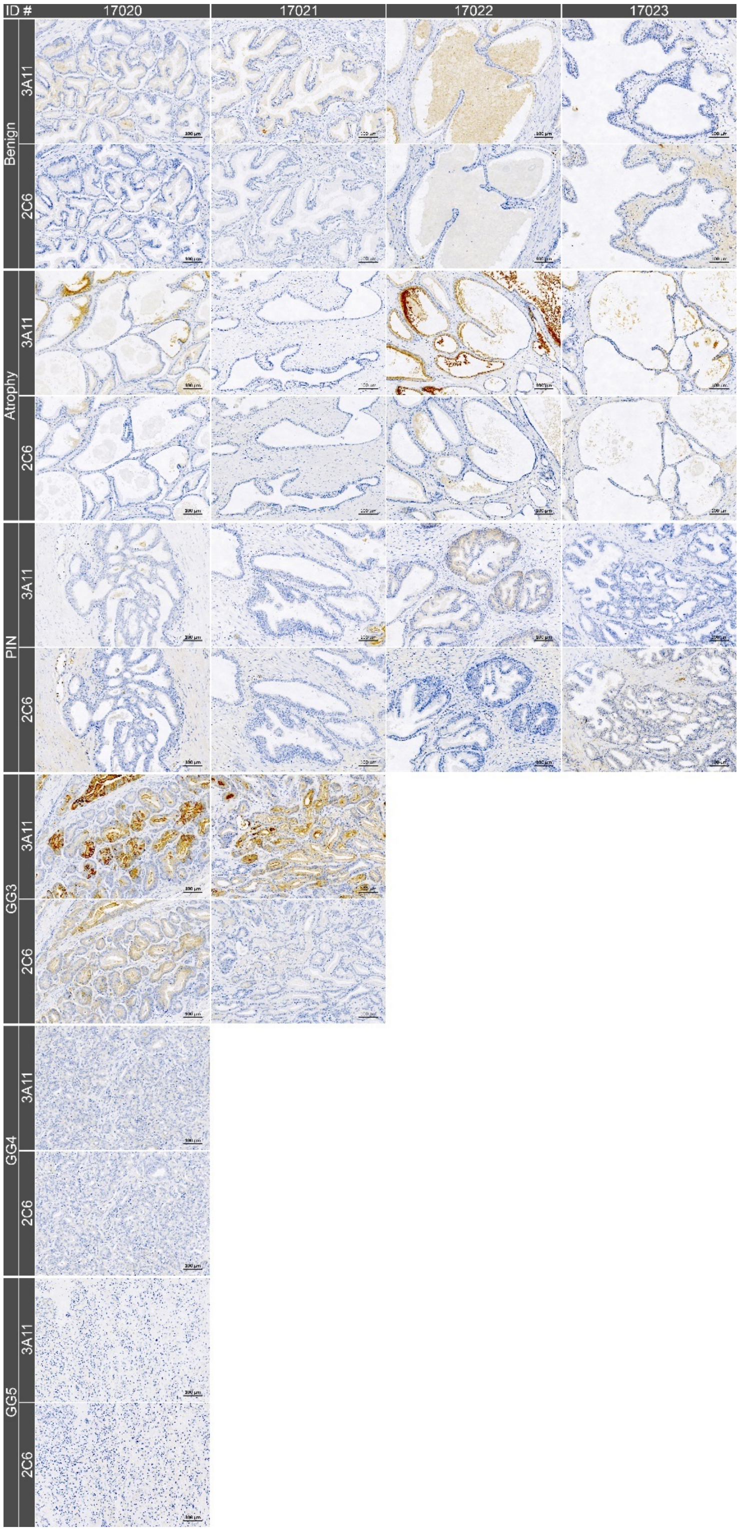

Figure S4. Additional immunohistochemistry staining of human prostate tissue sections using Synt-3A11 (0.13 ng/mL) and Synt-2C6 (0.26 ng/mL).
